# Fabrication of Bendable Circuits on a Polydimethylsiloxane (PDMS) Surface by Inkjet Printing Semi-Wrapped Structures

**DOI:** 10.3390/ma9040253

**Published:** 2016-03-30

**Authors:** Jiazhen Sun, Jieke Jiang, Bin Bao, Si Wang, Min He, Xingye Zhang, Yanlin Song

**Affiliations:** 1 School of Chemistry and Environment, Beihang University, Beijing 100191, China; jiazhensun@buaa.edu.cn (J.S.); baobin@iccas.ac.cn (B.B.); 2 Key Laboratory of Green Printing, Institute of Chemistry, Chinese Academy of Sciences, Beijing Engineering Research Center of Nanomaterials for Green Printing Technology, Beijing National Laboratory for Molecular Sciences, Beijing 100190, China; jiangjieke@iccas.ac.cn (J.J.); wangsi@iccas.ac.cn (S.W.); heminyiwen@iccas.ac.cn (M.H.); zhangxy@iccas.ac.cn (X.Z.)

**Keywords:** inkjet droplet, precured PDMS, depositing morphology, bendable circuit

## Abstract

In this work, an effective method was developed to fabricate bendable circuits on a polydimethylsiloxane (PDMS) surface by inkjet printing semi-wrapped structures. It is demonstrated that the precured PDMS liquid film could influence the depositing morphology of coalesced silver precursor inkjet droplets. Accordingly, continuous and uniform lines with a semi-wrapped structure were fabricated on the PDMS surface. When the printed silver precursor was reduced to Ag nanoparticles, the fabricated conductive film exhibited good transparency and high bendability. This work presented a facile way to fabricate flexible patterns on a PDMS surface without any complicated modification or special equipment. Meanwhile, an *in situ* hydrazine reduction of Ag has been reported using the vapor phase method in the fabricating process.

## 1. Introduction

Recently, printed electronics have attracted great attention in various device fabricating areas, such as liquid crystal displays, solar cells, organic light emitting diodes, electroluminescent devices and touch screen panels [1,2,3,4,5,6]. By virtue of its transparency, flexibility and good biocompatibility, polydimethylsiloxane (PDMS) has played an important role in patterning functional materials and fabricating devices, such as transfer printing, microfluidics and implantable medical devices [7,8,9,10,11]. Combining conductive patterns with PDMS will provide a way to fabricate various high-performance devices, such as stretchable conductors, wearable devices and electronic skins [12,13,14,15,16], which require electrodes for control and detection. In previous research, lots of work has been devoted to fabricating conductive patterns on a PDMS surface [17,18,19,20,21], but when using these approaches it is commonly difficult to fabricate flexible circuits on the surface.

Comparing with conventional patterning technologies, such as lithographic printing, nano-imprinting and micro-contact printing [22,23,24,25,26,27], inkjet printing provides a direct depositing technique using liquid materials, which is low-cost, convenient, flexible and fast. Inkjet printing has been recognized as one of the most promising methods to fabricate patterns on various substrates [28,29,30,31,32,33,34,35]. However, it is still a challenge to inkjet print controllable patterns on a low-adhesive PDMS surface [36,37,38], because the coalesced inkjet droplets tend to become a larger droplet due to the sliding three phase contact line with a driving force of shape relaxation [39,40,41,42]. In the traditional method, there would need to be some modifications to the PDMS surface before inkjet printing, such as oxygen plasma treatment or grafting polymer [43,44]. However, these methods also could not produce an ideal bendable circuit on the PDMS surface.

In this paper, we reported a facile strategy to fabricate bendable circuits on a PDMS surface by inkjet printing semi-wrapped structures. Trifluoroacetic acid silver salt solution was used as the inkjet printing ink. The depositing morphologies of coalesced silver precursor inkjet droplets were observed on the PDMS precursor substrates with different heating times. The precured PDMS liquid film played a crucial role in adjusting the depositing morphologies of the coalesced inkjet droplets, which was different from the rigid solid substrate. When an inkjet droplet struck the precured PDMS surface, the viscoelasticity of the liquid film could influence the wetting and depositing behavior of the microdroplet [45,46,47,48]. Then the continuous and uniform lines with semi-wrapped structure could be fabricated with the appropriate heating time of the PDMS precursor. When the printed deposits were reduced to Ag nanoparticles (AgNPs) by hydrazine hydrate, the fabricated conductive film exhibited good transparency and high bendability. This work presented a facile way to fabricate flexible patterns on PDMS surface without any modification, such as oxygen plasma treatment or grafting polymer. Meanwhile, an *in situ* hydrazine reduction of Ag has been reported using the vapor phase method in the inkjet printing process.

## 2. Results and Discussion

### 2.1. Fabrication and Characterization of the Inkjet Printed Circuit on PDMS Surface

**Scheme 1:**
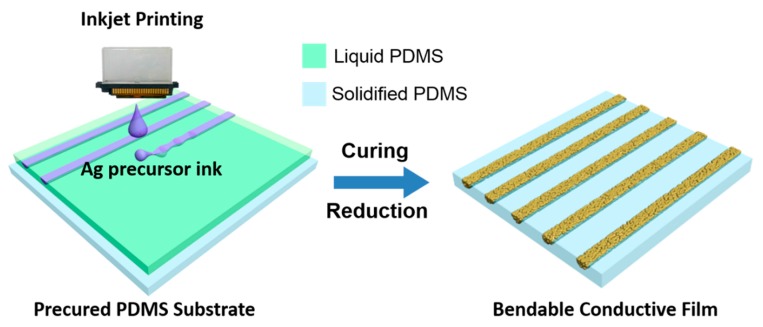
Schematic illustration of the fabricating bendable circuit on a precured PDMS liquid film by inkjet printing.

Scheme 1 shows the process of fabricating bendable circuit on PDMS surface by inkjet printing. A precured PDMS liquid film was used as the printing substrate. The Ag precursor solution was directly inkjet printed on the substrate. After heating the patterned substrate and reducing the inkjet printed deposits with hydrazine hydrate, the semi-wrapped conductive structures were directly fabricated on the surface of a PDMS film.

**Figure 1:**
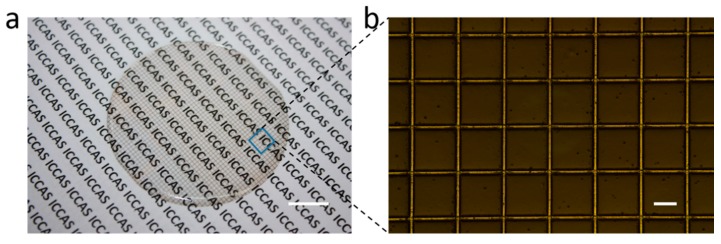
Structure of the inkjet-printed PDMS film. (**a**) A photograph of the patterned PDMS film, indicating its transparency. Scale bar 1 cm; (**b**) Magnified optical microscope observation of the inkjet-printed grid on PDMS surface. Scale bar 500 μm.

As shown in Figure 1a, the precise conductive grid was fabricated on the PDMS surface by inkjet printing and *in situ* vapor phase reduction. Meanwhile, the patterned PDMS film exhibited good transparency with the background. Figure 1b demonstrates that the precise grid was fabricated on the PDMS surface, which was composed of continuous and uniform lines. It indicated that this method could provide a route to fabricate circuits on the PDMS surface on a large scale without any complicated process, which will be of great significance for practical application.

### 2.2. Depositing Morphologies of Coalesced Inkjet Droplets on the Precured PDMS Liquid Film

**Figure 2:**
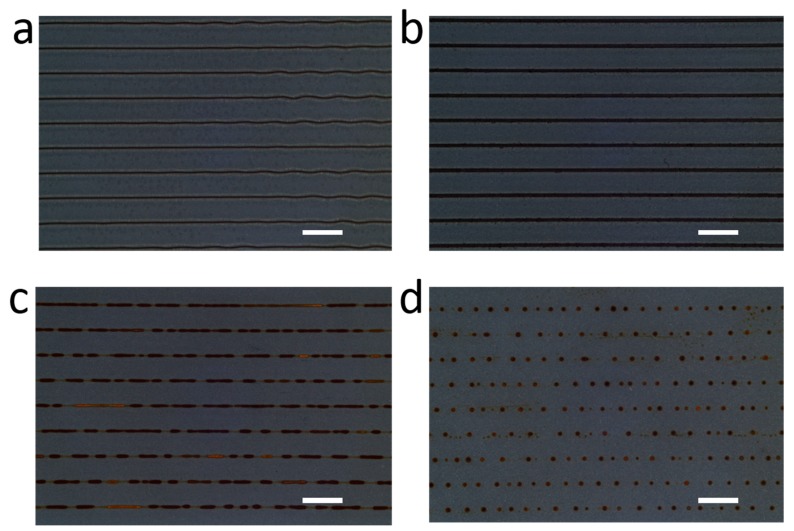
Depositing morphologies of coalesced inkjet droplets with different precured PDMS substrates. (**a**–**d**) Optical images of the inkjet printed patterns on the precured PDMS substrates with the heating time at 80 °C, 0 min, 10 min, 20 min and 30 min, respectively. Scale bar: 500 μm. When the ink was printed on an uncured PDMS precursor substrate, uniform lines were obtained. With 10 min heating time, the printed ink droplets also could maintain the uniform lines. When the heating time was increased to 20 min, the printed patterns changed to form periodical bulges. When the PDMS precursor substrate was heated for 30 min, the printed patterns tended to form a large discontinuous deposit.

A PDMS surface is a low adhesive surface, so a droplet slips and moves easily on it. Usually a complex pretreatment is required to inkjet print controllable patterns on such a surface, because there will be a force driving shape relaxation of the coalesced droplets when releasing the surface energy. As the viscoelasticity of the precured PDMS liquid film could influence the wetting and depositing behaviors of the microdroplet, the PDMS precursor substrates with a varied heating time were used to adjust the depositing morphologies of coalesced inkjet droplets. The optical images in Figure 2a–d shows that there were two typical depositing morphologies of the coalesced inkjet droplets: uniform line structure and bulged line structure. Figure 2a,b shows that short heating time of the PDMS precursor substrate resulted in a uniform line structure, whereas the bulged line structure was generated with relative long heating time for the PDMS precursor substrate (Figure 2c,d). To fabricate uniform lines by inkjet printing, matching surface tension between the liquid substrate and the printing ink is crucial. In our experiment, ethanol as the solvent was used to control the surface tension of ink for matching with the liquid PDMS substrate. In that case, the surface tension of ethanol (about 21.8 mN/m) is close to the liquid PDMS substrate (about 21.6 mN/m). Uniform lines could thus be inkjet printed on the liquid PDMS film. Meanwhile, the viscoelasticity of the precured PDMS substrate could also contribute to fabricating controllable patterns of the coalescing inkjet droplets. This would provide an opposing force to resist the sliding of the three-phase contact line (TCL) of the inkjet droplet in the shape-relaxing process to fabricate a uniform line [47,49]. By increasing the heating time of the PDMS precursor, the viscoelasticity of the precured PDMS would drop to a low value. Therefore, it could be determined that the heating time of PDMS precursor substrate greatly influenced the depositing morphologies of the coalesced inkjet droplets. As the TCL of the inkjet droplet slides during evaporation on the low-adhesive PDMS surface, it is a difficult task to directly inkjet print on a cured PDMS surface [50].

**Figure 3:**
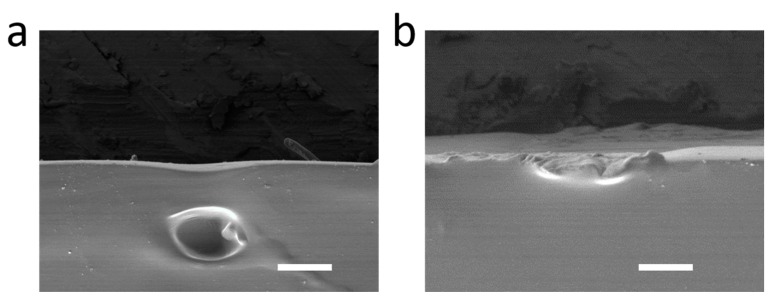
The location of the printed structure on the PDMS surface. (**a**) Side view SEM image of the printed line corresponding to the optical image in Figure 2a. The deposits were wrapped in the PDMS; (**b**) Side view SEM image of the printed line corresponding to the optical image in Figure 2b. The deposits were semi-wrapped on the PDMS surface. Scale bar: 20 μm.

In order to confirm the location of an inkjet-printed structure on the PDMS substrate, the SEM images of corresponding cross sections were observed, which exhibited the continuous and uniform depositing morphologies in Figure 2a,b. Figure 3a shows the cross section of the printed line with the uncured PDMS substrate. It was observed that the viscous liquid substrate wrapped the inkjet-printed deposits into the PDMS. The printed structure did not exist on the PDMS surface. Figure 3b shows the cross section of printed line on the PDMS precursor substrate after heating for 10 min. It could be observed that the printed structure existed on the PDMS surface, and it could also be partially wrapped into the precured PDMS substrate. Meanwhile, the semi-wrapped structure could contribute to the flexibility of the printed pattern and the gathering of printed materials. Therefore, the appropriate heating time of PDMS precursor substrate could be used to adjust the depositing morphology of inkjet droplets for fabricating continuous and uniform lines with a semi-wrapped structure on the PDMS surface. The optical images of printed deposits on the precured PDMS substrate with different focal planes also could indicate the location of an inkjet-printed structure (Figure S1). In the above process, when one droplet impacted the viscoelastic liquid substrate, the ejection of an inkjet droplet provided kinetic energy (Ek) and gravity provided potential energy (Ep). The viscoelastic film would provide a dissipating energy (Ed), which was used to maintain a stable state: Ek + Ep = Ed. When the liquid substrate has a short heating time, the high fluidity and low mechanical strength would cause the inkjet droplet to sink into the liquid film. Increasing the heating time, the fluidity of the liquid substrate would decrease and the mechanical strength of the liquid substrate would be enhanced. Once the viscoelasticity of the liquid PDMS film was adjusted to a proper value, the inkjet droplet would deform the surface of the viscoelastic PDMS film due to the impact of the droplet. A local deformation would form at the TCL of inkjet droplet, and the deformed viscoelastic PDMS surface hardly moved as the droplet evaporated. Thus a semi-wrapped structure could be fabricated with the solute precipitated from the inkjet droplet. Meanwhile, the ink concentration determined the shape of the deposit in the microgroove. With the evaporation of inkjet droplet, the density of precipitated silver salt was high enough to settle down with gravity. If the amount of deposit was too little, the deposit would just exist in the bottom of the microgroove. We have shown the printed result with a low ink concentration (3 wt %) in Figure S2. In our experiment, when the liquid PDMS film was precured with heating for 10 min, the silver salt ink with 15% mass could fill in the microgrooves.

### 2.3. In Situ Hydrazine Reduction of Ag with Vapor Phase Method

**Figure 4:**
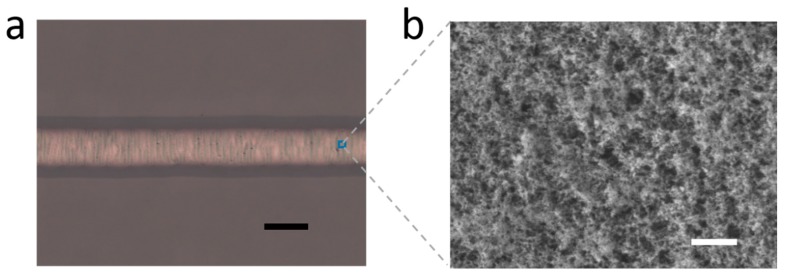

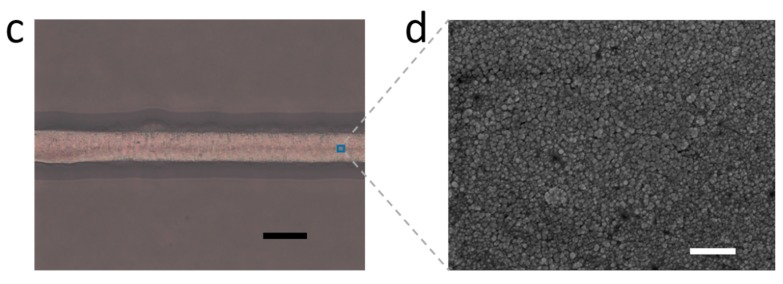
The structural transformation of the printed deposits after reduction with hydrazine hydrate. (**a**) Optical images of printed deposits before reduction; (**b**) magnified SEM image of the printed deposits before reduction; (**c**) Optical images of printed deposits after reduction; (**d**) magnified SEM image of the printed deposits after reduction, indicative of the AgNPs generation. (**a**,**c**: scale bar: 50 μm); (**b**,**d**: scale bar: 500 nm).

For printed electronics, metal nanoparticle ink is commonly used due to the high conductivity [51]. The ink consists of metal NPs and a carrier liquid solvent. For practical application, the nanoparticles need specifically designed surface properties which allow them to be stably dispersed in an appropriate solvent [52]. The polymer capping layers would be employed in the formulation of metal nanoparticle inks to prevent particles from aggregation and precipitation. However, the inkjet printing nozzle would be easily clogged with metal nanoparticle ink composed of highly volatile solvents. In this work, we used ethanol as the main solvent to match the surface tension. As ethanol is highly volatile, we used the trifluoroacetic acid silver salt as the solute of ink for good jettability [53]. After the ink was inkjet printed on the substrate, the solvent would evaporate and the Ag precursor was deposited on the substrate. Then the printed PDMS film was placed in a desiccator together with hydrazine hydrate. The desiccator was vacuumized for 2 min, and placed in an 80 °C oven for two hours. The vapor phase of hydrazine hydrate could provide good contact with the Ag salt in the thermal environment with a low pressure. As shown in Figure 4a–d, the Ag precursor was reduced to AgNPs. Particle size of the AgNPs was about 50–100 nm, which is shown in Figure S3. Comparing to the solution immersion method, this method of vapor phase reduction is a mild way to generate a conductive structure.

### 2.4. Measurement of Transparency and Conductivity

**Figure 5:**
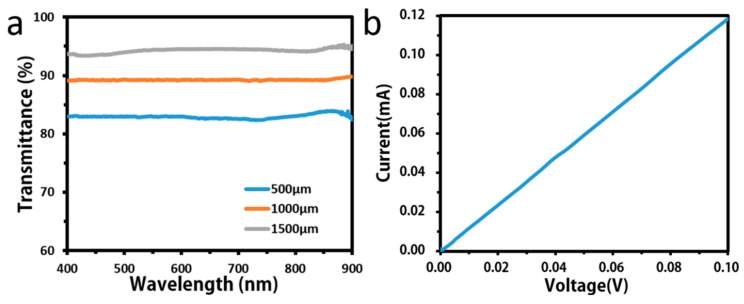
Transmittance spectra and current–voltage curve of the printed PDMS film after reduction. (**a**) The transmittance of the patterned PDMS with different grid spacings; (**b**) the printed circuits exhibited linear increase of the current when the sweeping voltage increased, indicating good ohmic contact among AgNPs.

Transparency is an important property for the applications of PDMS. As shown in Figure 5a, the transmittance of the printed PDMS film with different grid spacings showed good transparency after reduction in the visible spectra. The inkjet-printed line width was a crucial factor, which would influence the transmittance of the patterned PDMS film. The transmittance could be further improved by decreasing the printed line width while controlling the semi-wrapped structure or using other conductive materials [54]. Furthermore, the resistivity of reduced structure was measured as 2.93 × 10^−6^ Ω·m (the average of five independent measurements) by testing the conductivity of a certain segment. The I-V behavior of the printed circuit is shown in Figure 5b. An independent resistance test of the different reduced lines was also carried out, and the Ag lines showed good repeatability of conductivity (Figure S4).

### 2.5. Conductive Test of the Bent Semi-Wrapped Circuit

**Figure 6:**
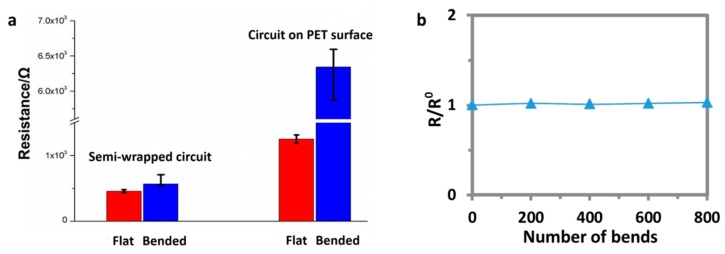
The resistance change of the bent semi-wrapped circuit. (**a**) The conductive comparison of the semi-wrapped circuit and circuit on PET surface before and after the bending; (**b**) The resistance change of the semi-wrapped circuit with repeated bending, R, R° indicate the resistance of the semi-wrapped circuit before and after the bending, respectively.

In this work, the reduced AgNPs were semi-wrapped into the PDMS film. As the semi-wrapped AgNPs were encapsulated by PDMS, the fabricated silver circuits could be used to resist bending without apparent resistance increase [55]. As a comparison, circuits inkjet printed on a PET surface using the Ag precursor ink were tested as the control sample. Then the two kinds of printed circuits were bent to 120°–150°, and the resistivity of printed circuits before and after the bending was measured by testing the I–V curve of the bent segment. Figure 6a shows that the resistance between the semi-wrapped circuit and circuit on PET film was not significantly different before the bending. When the semi-wrapped circuit was bent to 120°–150°, the circuit remained conductive with only a little resistance change between 200 ± 10 Ω. While the circuit on PET film was bent to 120°–150°, the resistance increased quickly and could not recover when the PET was flattened out. The stable conductivity of the semi-wrapped circuit resulted from the protection of the encapsulation structure. Then, the resistance after repeated bending was tested, comparing with the resistance of the sample without bending. Figure 6b shows that there was no apparent resistance increase after the semi-wrapped circuit was bent 800 times. After the bending, an LED was still lit with the bent film as a conductive unit (Figure S5), which demonstrated the excellent bendability of the fabricated circuits.

## 3. Experimental Section

### 3.1. Preparation of Printing Ink

The trifluoroacetic acid silver salt with 15% mass percent (Sigma-Aldrich) was dissolved in water/ethanol mixed solvent with volume ratio 10:90 (Sinopharm Chemical Reagent Co. Ltd., Beijing, China) and was used as the printing ink. The viscosity of the ink was 1.409 mPa·s. The surface tension of the ink was 23.8 mN/m. The density of the ink was 0.962 g/cm^3^. The solution was injected into an ink cartridge for use as printing ink.

### 3.2. Preparation of the Precured PDMS Substrates

PDMS base was mixed with a curing agent in the proportion of 10:1 by weight to make PDMS precursor (Sylgard 184, Dow Corning, Midland, MI, America). The precursor was put into a centrifuge to remove air bubbles (1000 rpm, 2 mins), then it was put onto a hydrophobic silicon wafer by spin-coating at 800 rpm for 20 s. The height of the liquid PDMS film was about 150 μm. Subsequently, the PDMS precursor was precured in an 80 °C oven for a given time and used as the printing substrate.

### 3.3. Inkjet Printing Silver Precursor Ink

The inkjet printing process was performed via a Dimatix Materials Printer (FUJIFILM DMP-2800 series, Tokyo, Japan) with one 25 μm nozzle and 10 μm inkjet droplet distance controlled with the Dimatix Drop Manager software. The environment temperature of the inkjet printing was 25 °C. A single nozzle was used during the printing process. The printing frequency was 5.0 kHz. A customized waveform was utilized with piezoelectric maximum 30 V and pulse width 8.5 μs.

### 3.4. Reducing the Printed Patterns

The printed patterns was placed in a desiccator together with 25 mL hydrazine hydrate (Sinopharm Chemical Reagent Co. Ltd., Beijing, China). The desiccator pumped air for 2 min and was then placed in an 80 °C oven for two hours.

### 3.5. Electrical Measurement

The electrical measurement was characterized under ambient conditions, using a semiconductor system (Keithley 4200-SCS, Keithley Instruments Inc, Cleveland, OH, USA) and an analytical probe station (Suss PM5). Two platinum conical probes were placed to contact the printed pattern, then applied impressed voltage to detect the corresponding current. The cross-sectional area was measured according to the scanning electron microscope (SEM) images. The resistivity ρ was calculated from the resistance *R*, the length *L*, and the cross-sectional area *A* of the measured conductive segment, using ρ = *R*·*A*/*L*.

### 3.6. Characterization

The structures of the printed patterns were investigated using an SEM (JSM-7500, JEOL Ltd, Tokyo, Japan). Optical micrographs were acquired with an optical microscope (Olympus MX40, Olympus Corporation, Tokyo, Japan). The transmittance of the printed PDMS conductive film was measured using a UV-Vis spectrophotometer (Shimadzu, UV 2600, Kyoto, Japan).

## 4. Conclusions

In summary, we demonstrated that a precured PDMS liquid substrate could be used to control the depositing morphologies of coalesced Ag precursor inkjet droplets. Then, semi-wrapped lines were fabricated on the PDMS surface. After the printed deposits were reduced to AgNPs by the *in situ* vapor phase reduction, a conductive film with good transparency and high bendability was fabricated. This work will be of significance for fabricating flexible circuits and interconnections in printed electronics.

## Supplementary Materials

The following are available online at www.mdpi.com/1996-1944/9/4/253/s1.
