# Supplementary material for: Fabrication of Bendable Circuits on a Polydimethylsiloxane (PDMS) Surface by Inkjet Printing Semi-Wrapped Structures
Source: Materials (Basel). 2016 Mar 30;9(4):253. doi: 10.3390/ma9040253 (PMC5502917; doi:10.3390/ma9040253)
Supplement: Supplementary file 1 [file materials-09-00253-s001.pdf]

Supplementary Materials: Fabrication of Bendable Circuits on a Polydimethylsiloxane (PDMS) Surface by Inkjet Printing Semi-Wrapped Structures

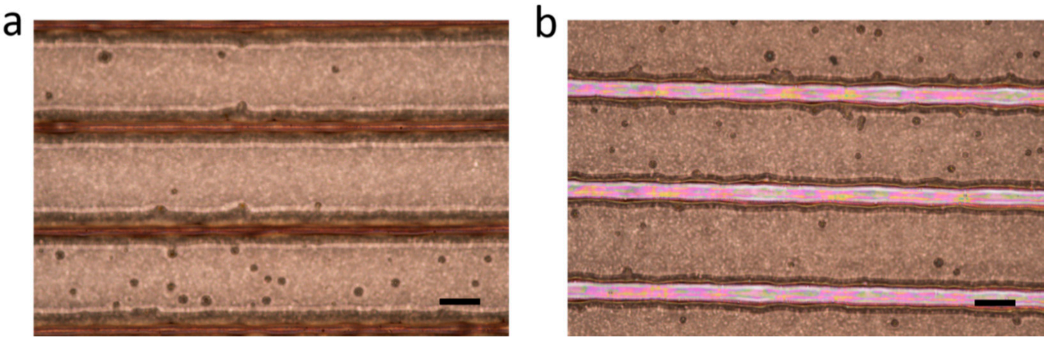

Figure S1. Optical images of printed deposits on the precured PDMS substrate with different focal planes. (**a**) The inkjet printed patterns on the precured PDMS substrate after heating for 0 min, at 80 °C; (**b**) The inkjet printed patterns on the precured PDMS substrate after heating for 10 min, at 80 °C. Scale bar: 100 μm.

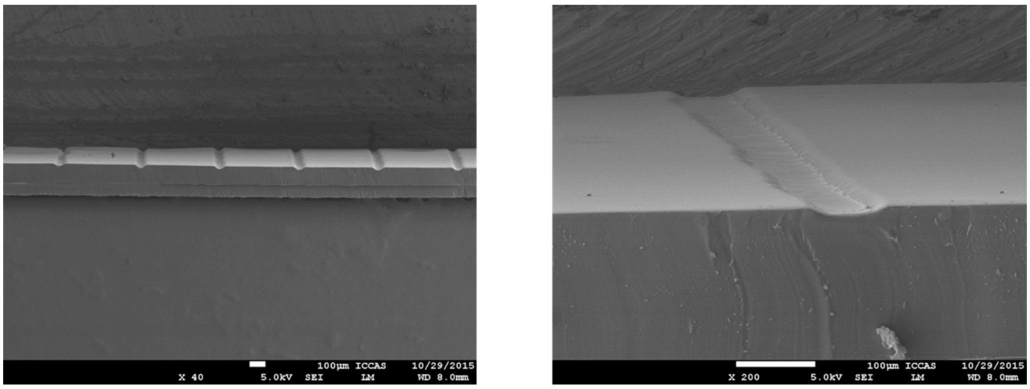

Figure S2. SEM images of printed deposits on the precured PDMS substrate with a low ink concentration (3 wt. %).

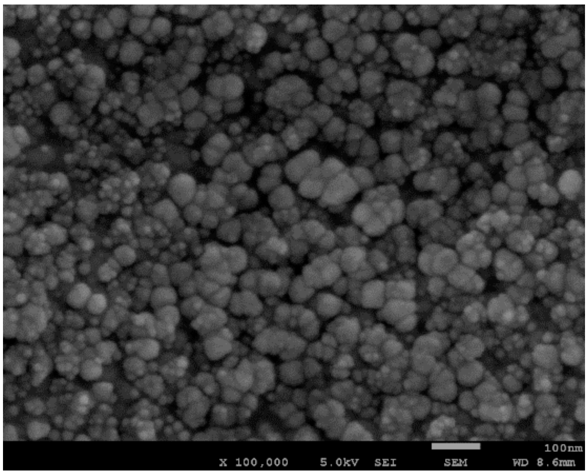

Figure S3. Magnified SEM image of the printed deposits after reduction, indicating that the particle size of the reduced AgNPs was about 50–100 nm.

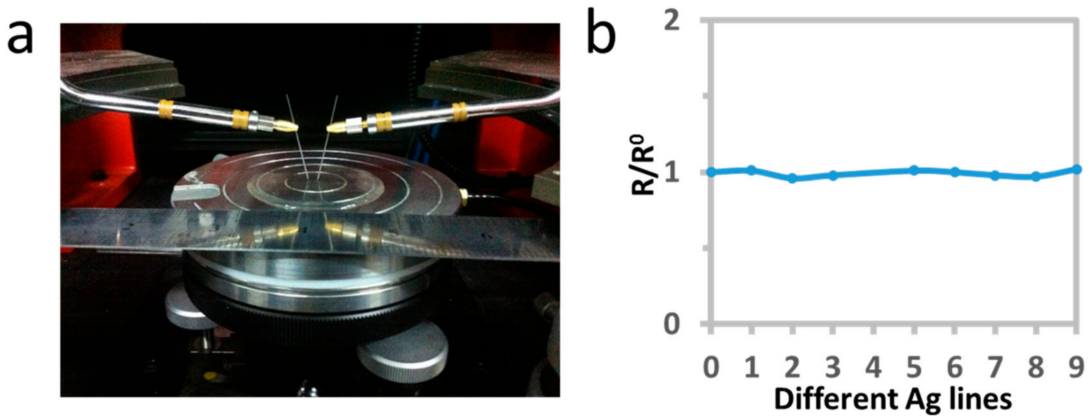

Figure S4. The conductivity of the different Ag lines (**a**) Photograph of the equipment and process of electrical measurements of the fabricated circuits; (**b**) The resistance change of the different Ag lines with a certain length, with R and R0 indicating the resistance of one initial Ag line and the others, respectively.

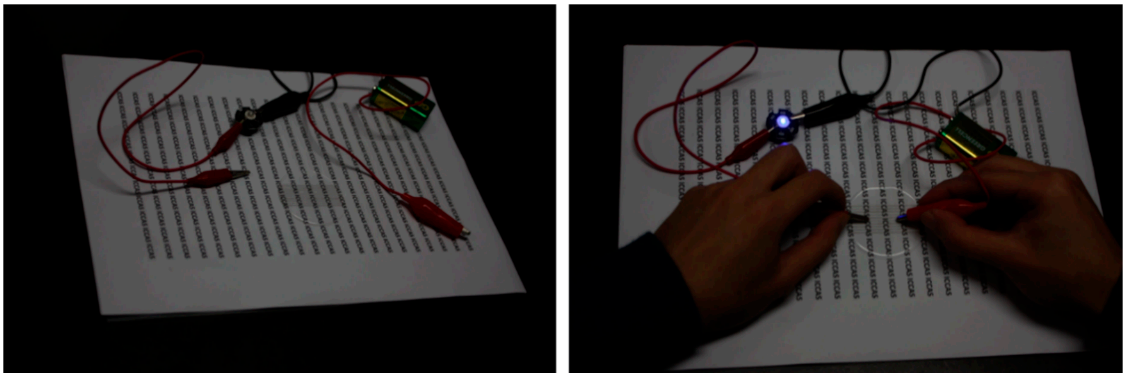

Figure S5. Demonstration of the bendability of the fabricated circuits. After bending 800 times, an LED was still lit with the bent film as conductive unit.
